# Inclusive Insurance, Income Distribution, and Inclusive Growth

**DOI:** 10.3389/fpubh.2022.890507

**Published:** 2022-04-14

**Authors:** Lili Zheng, Ya Su

**Affiliations:** School of Insurance, Central University of Finance and Economics, Beijing, China

**Keywords:** inclusive insurance, inclusive finance, income distribution, inclusive growth, balanced development, urban-rural dualistic economic structure, anti-poverty mechanism

## Abstract

Financial inclusion, whereby all adults have effective access to financial products, including insurance, has increasingly become a global priority, particularly in low and middle income economies. This study matches the measured development level of inclusive insurance in Chinese provinces with China Family Panel Studies (CFPS) data and evaluates the impact of inclusive insurance on income distribution and inclusive growth. The findings support that inclusive insurance has a positive impact on income distribution and inclusive growth. The effect is more pronounced in eastern areas, rural areas and low-income households. The policy shocks and instrumental variables introduced prove the robustness of the results. PSM-DID test indicate that the inclusive insurance policy has a significant positive effect on income distribution. Alternative measure of inclusive insurance and GMM test with instrumental variables indicate that the results are robust. Additionally, we also find that there is a threshold effect on the impact of the inclusive insurance on income. When the universal insurance index exceeds the threshold value, the promoting effect on income is enhanced.

## Introduction

“Inclusive finance” refers to a financial system that provides reasonable, convenient, and safe financial services to all segments and groups of society, with wide access to financial services and no price or non-price barriers (1). Non-bank financial institutions, especially insurance companies, play a very important role in inclusive finance (2, 3). In the indicator system for inclusive finance, Klapper et al. (4) used indicators related to health insurance and agricultural insurance, while Ambarkhane et al. (5) considered insurance-related indicators of the three dimensions of demand, supply and infrastructure. Sun et al. (6) argued that inclusive insurance is a collective term for various market-based policies that aim to maximize the benefits of disadvantaged groups. The purpose of inclusive insurance is to provide the insurance services needed by vulnerable groups such as rural households, urban low- and middle-income households, and small and medium-sized enterprises based on the principle of equal opportunity requirements and commercial sustainability (6–9). Based on the purpose of inclusive insurance, it can be seen that vulnerable groups, equal opportunity, fairness, security, and an incomplete market are the key words of inclusive insurance.

The development of inclusive finance was a major strategic deployment made at the Third Plenary Session of the 18th CPC Central Committee in China. At present, the rapid development of inclusive insurance is playing an important role in medical and health care, social security, employment, small and microenterprise development, agricultural production and other aspects of guaranteeing the livelihood of people in China (10). In terms of medical care, in 2020, 23 provinces, 82 regions and 179 cities in China launched Huimin insurance. On the basis of serious illness insurance for urban and rural residents, Huimin insurance has increased the reimbursement for special drugs for major diseases and delivery services, and it has increased the proportion of compensation, covering more than 40 million participants.^1^ In terms of pension, in 2019, the General Office of the State Council issued the Opinions on Promoting the Development of Pension Services, stating that it is important to carry out reverse mortgage pension insurance business for elderly individuals and to encourage insurance companies to design accident insurance suitable for elderly individuals. The inclusive features of these two types of insurance can guarantee the basic living needs of elderly people and contribute to the establishment of a multilevel and sustainable old-age security system in China. Microinsurance is also a form of inclusive insurance, and micro loan guarantee insurance helps solve the problems of difficult and expensive financing for small and micro enterprises. In terms of agricultural production, in 2007, China launched a pilot scheme of subsidizing agricultural insurance premiums based on the central government, and it proposed the basic policy of agricultural insurance operation consisting of “government guidance, market operation, independent and voluntary, and coordinated promotion.” Huimin insurance, reverse mortgage pension insurance, accident insurance for elderly people, micro loan guarantee insurance, and agricultural insurance are typical representatives of inclusive insurance, and they play an important role in many important industries and key areas related to people's livelihood (10). With the development of inclusive insurance, the socioeconomic effects of inclusive insurance have stimulated academic discussions. As a special mechanism with social inclusion, inclusive insurance can not only effectively share the pressure of mitigating social risks (11) but also improve the availability of insurance by reducing the supply cost of insurance services, reducing the cost of purchasing insurance, and improving the level of social security (12).

Income distribution and inclusive growth are also key issues emphasized in China's development. The Sixth Plenary Session of the 19th CPC Central Committee that was just held stressed “implementing the new development concept, building a new development pattern, promoting high-quality development, comprehensively deepening reform and opening up, promoting common prosperity, and insisting on safeguarding and improving people's livelihood in development.” The idea that “the income distribution system must be perfected” was mentioned in “Some Major Issues of the National Medium- and Long-Term Economic and Social Development Strategy of China” (13). It can be seen that both income distribution and inclusive growth are issues that need attention, and inclusive insurance has an important role to play in both income distribution and inclusive growth. Inclusive growth not only pays attention to the mode and speed of growth but also pays more attention to improving equal access and opportunities, and it emphasizes the importance of “social exclusion and lack of rights” (14). The concept of inclusive growth opposes the notion that the achievements of development are enjoyed only by some or even a few people. It requires all people to benefit from development, sharing the results of development (15), and focuses on access to benefits among groups (16, 17), which coincides with the concept of inclusive insurance.

In this context, it is particularly important to systematically explore the impact of inclusive insurance on income distribution and inclusive growth. The innovation of our research is reflected in the following: First, most studies on inclusive insurance have focused on its definition and index measurement and on the testing of its determinants. Based on the construction of an inclusive insurance index, we are the first to discuss and explore the effect of inclusive finance on income distribution and inclusive growth as well as the mechanism, providing theoretical support and practical guidance for the design of an inclusive insurance policy framework. Second, most existing studies have explained the economic effects of inclusive insurance at the theoretical level, and empirical analysis has focused on the provincial and municipal levels. However, the economic environment in China may lead to deviations, including deviations between the micro action mechanism and the macro expected effect. We combine the measured level of inclusive insurance development in each province of China with China Family Panel Studies (CFPS) data to examine the impact of inclusive financial development on residents' income and income growth at the micro level and to extend the micro mechanism of the economic effect of inclusive insurance. Third, we analyze the “growth effect” and “distribution effect” of inclusive insurance development on residents' income in the same framework and then explores whether the income-increasing effect of inclusive insurance is heterogeneous, how the mechanism of inclusive insurance affects income distribution and inclusive growth, and how inclusive insurance affects the income gap and income growth through the heterogeneity of health, human capital and social capital. It also provides corresponding theoretical support and an empirical basis for the development of inclusive insurance.

## Related Literature and Research Hypotheses

### Inclusive Insurance

Inclusive insurance encompass many different approaches to reaching the unserved, underserved, vulnerable, or low-income populations in emerging markets with appropriate and affordable insurance products. These range from microinsurance for people with very little disposable income to new products and services for an emerging middle class around the globe who have not been served by traditional insurance. The World Bank (18) estimates that 1.2 billion (20%) people worldwide live on < $1 a day (extreme poverty) and that another 1.8 billion (30%) live on < $2 a day (moderate poverty). Among these people, only 1–3% have access to insurance products (19), and these households remain highly vulnerable to risks from a range of issues, such as disease, livestock losses, and catastrophic weather events (20). Research on inclusive insurance has focused on microinsurance. Microinsurance has been defined as “any form of protection against risks that is designed for and accessed by low-income people, provided by different categories of carriers but operating on basic principles of insurance and funded by premiums” (21). Heenkenda (22) pointed out that microinsurance is an inclusive insurance service for poor and low-income people. Microinsurance targets groups in the unconventional economy that are not served by mainstream commercial insurance. Although low-income groups face the same risks and shocks as traditional insurance clients, they are more vulnerable due to resource and knowledge constraints (1, 23), and they have less ability to cope when they suffer losses. Microinsurance has been proven to be beneficial for low-income people. It is the best choice for low-income people to build confidence and restore wealth when risks occur (24). Microinsurance matches its premiums and benefits with the needs of these groups (20, 25, 26). Considering that the premium matches the possibility of risk occurrence, it can protect customers from specific risks (27). Inclusive insurance extends insurance coverage to all those who do not receive traditional insurance services, including the lower and middle classes, while placing special emphasis on vulnerable and low-income people (28). Roa et al. (29) argued that inclusive insurance mainly includes microinsurance, which is characterized by low cost, simple terms, basic coverage and easy access, and brings obvious benefits to low-income people. Insurance institutions such as Allianz and AXA that have developed microinsurance products consider microinsurance to serve emerging consumers or clients, while Latin America uses the concept of mass insurance or popular insurance more often, the International Labor Organization (ILO) uses the concept of impact insurance, and China uses the concept of inclusive insurance. Some scholars in China have discussed the model and development of inclusive insurance. Wang and Yin (30) discussed six models of inclusive insurance, including microinsurance, health insurance, agricultural insurance, small business insurance, credit guarantee insurance and insurance investment. Sun et al. (6) measured the development level of inclusive insurance in China. On the basis of measuring the development level of inclusive insurance, Yin et al. (31) showed that the development level of inclusive insurance in the western region of China was higher than that in the eastern region from 2007 to 2017.

Scholars' discussion of the economic effects of inclusive insurance mainly focuses on poverty reduction effects. Zhang and Jiang (32) found that a one standard deviation increase in the village inclusive insurance index will reduce the possibility of rural households falling into poverty by 33.24%. They pointed out the significant effect of inclusive insurance on reducing vulnerability to poverty. Xu and Chen (33) argued that inclusive insurance is effective in reducing the incidence of poverty in a region and adjacent areas. More studies are based on the microinsurance or agricultural insurance perspective, which argues that agricultural insurance can trigger a virtuous cycle of increased agricultural investment and increased farmers' income (26), with a promoting effect on farm household business income (34–36) and improving the stability and sustainability of farmers' income (37, 38). Hamid et al. (39) argued that microhealth insurance can reduce poverty by improving the health status of poor individuals and reducing the health risks of insured individuals. Other scholars highlight the role of insurance under the system of financial inclusion. Ma and Du (40) showed that the role of insurance is greater than that of banks in explaining changes in poverty alleviation based on the three-dimensional indices of inclusive finance (macro, banking and insurance).

### Inclusive Insurance and Income Distribution

Income distribution has always been one of the economic and social issues of greatest concern to scholars. Some scholars have studied the impact of insurance on income distribution. Some scholars believe that the adjustment effect of insurance on the income gap is not obvious, and there is inequity in insurance. Jin et al. (41) found that the income redistribution effect of China's basic medical insurance system is negative. The medical expenditure and medical insurance reimbursement of low-income groups with poorer health status are significantly lower than those of high-income groups. After simulation evaluation, Zhong (42) found that there was a serious endogenous lack of fairness in the basic endowment insurance of employees in Chinese enterprises. He and Sato (43) found that China's urban social security system lacks progressivity in contributions and pro-poor benefits; thus, the redistributive effects have difficulty offsetting widening income inequality. The study by Li et al. (12) found that the inequality in pensions due to differences in policy coverage and institutional parameters can have a significant exacerbating effect on the income inequality of older households. Guo et al. (44) argued that there is more severe agricultural insurance exclusion in China, which has a significant negative impact on farmers' income. Tan and Zhong (45) found that the impact of the new rural cooperative medical system on the income equity of medical expenditure groups is significantly positive and that the compensation of low-income groups is higher than that of high-income groups. Different compensation modes regulate income distribution differently. Yao et al. (46) found that there are income-related inequities in the hospitalization service and medical insurance reimbursement level of basic medical insurance in China. Urban workers' medical insurance has a higher degree of equity, and rural residents' medical insurance has a poorer degree of benefit equity. Huang (47) argued that the poverty alleviation effect of urban health insurance policies has obvious heterogeneous characteristics across households with different incomes, especially for middle-income and high-income participating households.

Due to the economic development strategy and insurance system arrangement, the effective insurance supply (insurance institutions, insurance products, insurance services) is insufficient. Some residents are excluded from the traditional insurance system due to their low income level, poor risk resistance ability, small scale of production and operation, lack of credit information and other reasons, and they cannot avoid life and production risks through the insurance system. Inclusive insurance can correct the resource mismatch, and microeconomic individuals can effectively use a more balanced resource allocation to achieve self-development. Even among people with very little disposable income, insurance can be an important tool for managing risk and helping them mitigate the stress of illness, crop failure, natural disasters or loss of income due to the death of the head of household (4). The role of insurance in coping with natural disasters and developing resilience is well-established. Insurance provides an incentive for households to invest before shocks occur to avoid them or after shocks occur to mitigate them. For example, health insurance encourages low-income people to go to the doctor, thereby reducing mortality among this group. Farmers with insurance are often eligible for credit to buy high-yield, drought-resistant crop seeds, and these farmers can avoid selling assets to cope with losses even in the event of a drought. Insurance can also help households avoid drastic coping strategies when exposed to risk, such as taking children out of school. Inclusive insurance improves residents' access to insurance services and products by reducing the cost and increasing the accessibility of insurance services. On the one hand, by influencing savings behavior, inclusive insurance increases the wealth accumulation of vulnerable groups' families, increases investment capacity and optimizes production decisions, thus having a positive impact on income. On the other hand, more vulnerable households can avoid physical, life and property risks through the insurance system, avoid enormous fluctuations in income level, and improve the ability to resist risks. All these factors have an impact on the income of disadvantaged residents, thus optimizing income distribution. Therefore, the development of inclusive insurance can improve the operational efficiency of agriculture and the income level of rural residents, thus effectively reducing the income gap, the urban-rural gap and the regional gap. Research has found that microinsurance contributes to social protection and could be a tool for reducing the insurance gap for nearly 61% of uninsured low-income families in South Africa as a type of inclusive insurance (48).

Therefore, this study formulates Hypothesis 1: inclusive insurance contributes to the optimization of income distribution.

### Inclusive Insurance and Inclusive Growth

Inclusive insurance contributes to inclusive growth by providing more equitable and broad-based insurance services in response to “market failures” in the development of insurance from the perspective of service equity.

First, inclusive insurance, such as microguarantee insurance and medical insurance, can enable more effective risk management, mitigate the losses suffered by individuals and enterprises, protect affected families from major financial losses and help enterprises resume production in a timely manner, thus guaranteeing the continuity and stability of social reproduction. Inclusive insurance also contributes to the development of the rural economy and agriculture. By providing basic security for rural residents, it promotes the smooth progress of agricultural production and the stable development of the rural economy. Agricultural insurance and microcredit insurance establish rural financial risk compensation and decentralization mechanisms, which will help farmers expand their production scale, improve production efficiency and support the construction of modern agriculture.

Second, the economic growth effect of insurance development is realized through two types of functions, risk transfer and financial intermediation (49), and inclusive insurance can smooth the consumption of individuals and firms through risk transfer and payouts, encouraging them to shift their savings to investment (50–58). With equal opportunities and fairness, more residents can enjoy insurance services, which increase the risk transfer channels and residents' willingness to invest. Losses without insurance have negative externalities for individuals and families. This anxiety with regard to life, health and property can be ameliorated through the purchase of universal insurance and the promotion of the population's consumption activities (59). Insurance payouts or life insurance benefits can improve the risk tolerance of residents so that they plan consumption expenditures over a longer period of time; that is, residents can smooth their consumption activities over a longer period of time.

Third, inclusive insurance has a direct impact on rural social development. With the popularization of inclusive insurance in remote rural areas, on the one hand, hardware facilities in rural areas have been built, and the infrastructure represented by insurance infrastructure has been gradually improved. The production conditions, living conditions and life outlook of rural residents have been greatly improved. On the other hand, it can also microscopically improve the financial literacy and insurance awareness of rural residents, thus promoting inclusive development.

Therefore, this study formulates Hypothesis 2: inclusive insurance is conducive to inclusive growth.

## Methodology

### Data Sources

Our research uses household and provincial data. The household data are from the China Family Panel Studies (CFPS) conducted by the Institute of Social Science Survey of Peking University. Twenty-five provinces, municipalities and autonomous regions are covered, and survey data from the base period of 2010 and tracking data from 2012, 2014, 2016, and 2018 are included. The provincial data are panel data on all provinces, municipalities and autonomous regions from 2010 to 2020, all of which come from the China Statistical Yearbook, the statistical yearbooks of each province, city and autonomous region, the China Insurance Yearbook, the China Economic Net and the WIND database. The data were processed as follows: firstly, Samples aged 18–60 years old were selected; Secondly, the adult questionnaire and household questionnaire were matched, and samples with “don't know” and “not applicable” were treated as missing values. The missing values of important variables were excluded, and linear interpolation or the mean value was used to supplement or replace individual missing values. Lastly, the continuous variables involved were subjected to a 1% tailing process. The final sample is composed of 75,737 samples from 16,640 households.

### Variables

#### Independent Variable

China's Plan for Promoting the Development of Inclusive Finance (2016–2020) states that the inclusive finance indicator system should contain three dimensions: broad inclusiveness, the degree of specificity matching and commercial sustainability. Scholars have discussed the variables that should be included in inclusive finance (6, 31, 60–64), and they generally agree that inclusive insurance should have broad inclusiveness and insurance accessibility, including the degree of coverage of insurance services in terms of the population and space. Broad inclusiveness is the basic requirement of inclusive insurance. The degree of specificity matching is related to the principle of equal opportunity in the concept of inclusive insurance development, referring to support for those who are excluded from insurance. On this basis, considering the availability of data, we use three dimensions, accessibility, penetration and use efficiency, to construct the index of the development level of inclusive insurance. The premium growth rate, the number of insurance institutions per capita and the number of insurance institutions per 100^2^ km, representing the market scale, reflect the degree or proportion of consumers' exposure to insurance, which can measure the accessibility of insurance. Insurance density, insurance depth and the payout per capita can reflect the penetration level of insurance to a certain extent. The goal of insurance is protection; thus, the use efficiency of insurance is measured by the protection level and the payout level.

#### Income Distribution

Income distribution is an important explanatory variable, and CFPS survey data have detailed information on income and are comparable. The relative deprivation index (Rel) (65) has been used in many studies to analyze the income distribution of micro households. The relative deprivation of income refers to the sense of deprivation generated when poor families find that their income is at a disadvantage compared with the average income of the reference group, and it can reflect the change in the income gap at the family level (66). It reflects the inequality underlying the change in family income and has good properties with regard to fitting income distribution (67). Therefore, we use the Kakwani income Rel to measure income distribution. The specific measurement process is as follows: let Y represent the total sample and n represent the number of samples, and arrange the income of the sample households in ascending order so that the overall income distribution of the sample is Y = (y_1_, y_2_, …., y_n_); thus, the income inequality of household y_i_ is


(1) $$\begin{array}{l} RD\left(y,y_{i}\right)=\frac{1}{n\mu_{y}}\left(\overset{n}{\underset{j=i+1}{\sum }}\left(y_{j}-y_{i}\right)\right)=r_{y_{i}}^{+}\;\left(\mu_{y_{i}}-y_{i}\right) \end{array}$$

where μ_*y*_ is the mean income of all samples, μ_*y*_*i*__ is the mean income of households earning more than *y*_*i*_, $r_{y_{i}}^{+}$ is the percentage of the total sample whose income level exceeds *y*_*i*_, and the index is between 0 and 1.

#### Control Variables

In addition to inclusive insurance, other economic factors may have some impact on income distribution and inclusive growth. With reference to the existing literature (68–70), we control for individual factors, household factors and regional factors that affect income distribution and inclusive growth in our model. Individual characteristics include the gender, age, marital status, registration, education, health, and employment status of the household head. Household factors include financial assets, the debt situation, and access to information (71). Regional factors include the level of economic development, urbanization, fiscal revenue, the industrial structure, infrastructure, and the unemployment rate. Table 1 lists the descriptions of each variable.

**Table 1:** Model variable design.

**Variable classification**	**Variable measurement**	**Variable name**	**Variable meaning**	**Variable description**
Explained variables	Income	Inc	Household income	To ensure the comparability of data from different sample years, we adjusted income based on the consumer price index (CPI) published by the China Price Statistical Yearbook, and took 2012 as the base period, excluding the impact of price changes
	Income inequality	Rel	Relative deprivation index	Based on the Formula (2)
Explanatory variables	Contact	Ins-per	Number of insurance institutions per capita	Number of insurance companies (institutions)/total population of the province
		Ins-squ	Number of insurance institutions per 100 square kilometers	Number of insurance companies (institutions)/land area of the province
		Pre	Premium growth rate	Premium income of the province for the current year/premium income of the previous year
	Permeability	Den	Insurance density	Premium income/total population of the province ($/person)
		Dep	Insurance depth	Premium income/GNP of the province (%)
		Com	Payout per capita	Total insurance claims and payments/total population of the province
	Use efficiency	Sec	Coverage level	Total amount of insurance company coverage/total population of the province
		Comp	Payout level	Total insurance claims and payments/insurance premium income of the province
Control variables	Individual level	gen	Gender	Dummy variables, where male takes the value of 1 and female takes the value of 0
		age	Age	Actual age of the household head (years)
		mar	Marital status	Unmarried, divorced and widowed take the value of 0 and married (with spouse) takes the value of 1
		hou	Household registration type	Farmers take the value of 0; non-farmers take the value of 1
		edu	Years of education	Years of education completed
		hea	Health level	It is determined by the response to the question “What do you think of your health status?,” which respondents answer using a 5-point Likert scale. “Very healthy,” “quite healthy,” and “relatively healthy” take the value of 1 and the rest take the value of 0
		emp	Employment status	Employment take the value of 1; unemployment and withdrawal from the labor market take the value of 0
	Family level	fin	Financial assets	Financial assets include deposits, stocks, funds, bonds, financial derivatives, other financial products, and borrowings. If the respondent has any of these assets, the variable takes the value of 1 and 0 otherwise
		deb	Debt status	Outstanding debt mainly refers to debt related to formal financial institutions, which is determined based on the total amount of outstanding mortgage principal and interest and other outstanding loan amounts in the questionnaire. The questions are “How many yuan does your family still owe on the bank mortgage that has not been paid off?” and “In addition to the mortgage, how many yuan does your family still owe the bank that has not been paid off?”
		inf	Information acquisition	Response to the question “How important is the internet for you to obtain information" in the adult questionnaire.^a^ 1 means very unimportant, and 5 means very important
	Regional level	Dev	Regional economic development level	GDP per capita in the area where the household is located (10,000 yuan). According to Binkai and Yifu (72), the income gap between urban and rural areas and the level of economic development show a U-shaped law of first decreasing and then increasing
		Urb	Urbanization level	The urbanization rate is used to measure the urbanization level of a region in a certain year The amount of the urban population in each province and year/the total urban and rural population at the end of each year
		Fis	Fiscal revenue level	Expressed in terms of public fiscal revenue per capita
		Str	Industry structure	Share of the tertiary sector in GDP
		Tel	Infrastructure level	The number of landlines per capita is used as a proxy variable
		Une	Unemployment rate	Measured by the urban registered unemployment rate

a *The popularization of the internet, represented by large-scale broadband access, accelerates information transmission and reduces transaction costs, while creating favorable conditions for economic development, total factor productivity growth, and per capita income improvement (73)*.

### Model

#### Inclusive Insurance and Income Distribution

The effect of inclusive insurance on income distribution was tested using a fixed effect model designed as follows.


(2) $$\begin{array}{l} Rel_{it}=\beta_{0}+\beta_{1}II_{it}+\overset{N}{\underset{k=1}{\sum }}\beta_{k}X_{kit}+\emptyset_{i}+\varphi_{i}+\mu_{it} \end{array}$$

where Rel_it_ is the relative deprivation index, II_it_ is the level of inclusive insurance coverage, X_kit_ is the set of control variables, and μ_it_ is a random disturbance term.

#### Inclusive Insurance, Income Distribution, and Inclusive Growth

To further explore whether inclusive insurance can lead to inclusive growth, drawing on the study by Zhang et al. (74), the lagged term of income and the interaction term of the lagged term of income and the inclusive insurance index are added.


(3) $$\begin{array}{l} \begin{array}{c} Income_{it}=\alpha_{0}+\alpha_{1}\text{I}{\text{I}}_{\text{it}}+{\alpha_{2}Income}_{it-1}+\alpha_{3}\text{I}{\text{I}}_{\text{it}}*Income_{it-1}\\ \;+\sum_{k=1}^{N}\alpha_{k}X_{kit}+\emptyset_{i}+\varphi_{i}+\;\mu_{it} \end{array} \end{array}$$

where Income_it_ is the income of resident i in year t, α_0_ is the intercept term, ∅_*i*_ is the individual fixed effect, φ_i_ is the time fixed effect, and μ_*it*_ is the sum of the two-way fixed effects and the error term.

Clearly, when II_it_ = 0, the value of the target variable is


(4) $$\begin{array}{l} E\left(y_{it}|II_{it}=0\right)=\alpha_{0}+\alpha_{2}y_{it-1}+\overset{N}{\underset{k=1}{\sum }}\alpha_{k}X_{kit} \end{array}$$

When II_it_ = 1, the value of the target variable is


(5) $$\begin{array}{l} E\left(y_{it}|II_{it}=1\right)=\alpha_{0}+\alpha_{1}+\alpha_{2}y_{it-1}+\alpha_{3}y_{it-1}+\overset{N}{\underset{k=1}{\sum }}\alpha X_{kit} \end{array}$$

To facilitate the analysis of the relationship between inclusive insurance and inclusive growth, the model is simplified as follows:


(6) $$\begin{array}{l} E_{p}=E\left(y_{it}|II_{it}=1\right)-E\left(y_{it}|II_{it}=0\right)=\alpha_{1}+\alpha_{3}y_{it-1} \end{array}$$

Based on the above equation, the influence of II_it_ on y_it_ can be divided into two parts: α_1_ measures the effect of inclusive insurance II_it_ on target variable y_it_ when other conditions remain the same; α_3_y_it−1_ measures the heterogeneous influence of the income level in the previous period on the income level in the current period through the current period's inclusive insurance index: If α_3_ > 0, the higher the income level (the larger the y_it−1_), the more benefits from inclusive insurance; if α_3_ < 0, the lower the income level (the smaller the y_it−1_), the more benefits from inclusive insurance, indicating that the development of inclusive insurance in a region helps to raise the income level of low-income residents and alleviate income inequality.

Officially, when α_1_ + α_3_*y*_*it*−1_ > 0 and α_3_ < 0, we define that inclusive insurance improves the distribution of income and brings about inclusive growth. This is when inclusive insurance brings about growth in the target variable, on the one hand, and improves the distribution of the target variable, on the other hand. If only α_1_ + α_3_*y*_*it*−1_ > 0 but α_3_ > 0, then inclusive insurance leads to income growth, but it causes an increase in income inequality and therefore does not contribute to inclusive growth.

## Empirical Results

### Inclusive Insurance Index

Principal component analysis was used to synthesize multiple indicators of inclusive insurance into one index. All data were calculated based on the means, and the covariance matrix was used to reflect the differences in the degree of variation of each indicator, resulting in the eigenvalue, range of change, impact rate and cumulative impact rate of each indicator (see Table 2).

**Table 2:** Factor analysis of the variance contribution.

**Factor**	**Eigenvalue**	**Range of**	**Impact**	**Cumulative**
		**change**	**rate**	**impact rate**
Factor 1	4.03145	2.45603	0.5039	0.5039
Factor 2	1.57543	0.627158	0.1969	0.7009
Factor 3	0.948272	0.406895	0.1185	0.8194

As shown in Table 2, Factor 1 has the highest degree of influence, accounting for 50.39%. Three factors are extracted, with a cumulative contribution rate of 81.94%. Taking 80% as the standard of the accumulated load, three factors are selected as the basis of index synthesis, and their respective loadings are used as weights to calculate the correlation between each subdivision index and the factor to obtain the factor expression coefficient of each original variable. To better separate and strengthen the role of each factor and make the explanation of the original variable clearer, we use the method of varimax rotation to carry out orthogonal rotation of the factor matrix. Finally, three comprehensive factors are formed, each of which is related to the original variable. The factor scores of each variable are shown in Table 3.

**Table 3:** Factor analysis of the rotated components and score coefficients.

**Variables**	**Factor 1**	**Factor 2**	**Factor 3**
Ins-per	0.436	−0.0129	0.2947
Ins-squ	0.4095	−0.0046	0.4662
Pre	−0.0998	0.6081	0.3754
Den	0.3083	0.3567	−0.5478
Dep	0.4512	0.1156	−0.2618
Com	0.4589	−0.0652	−0.2512
Sec	0.3481	−0.0957	0.3497
Comp	0.0445	−0.6899	−0.0296

Based on the variance contribution of the table and the factor score coefficients in Tables 2, 3, the inclusive insurance index II was obtained.

**Figure 1:**
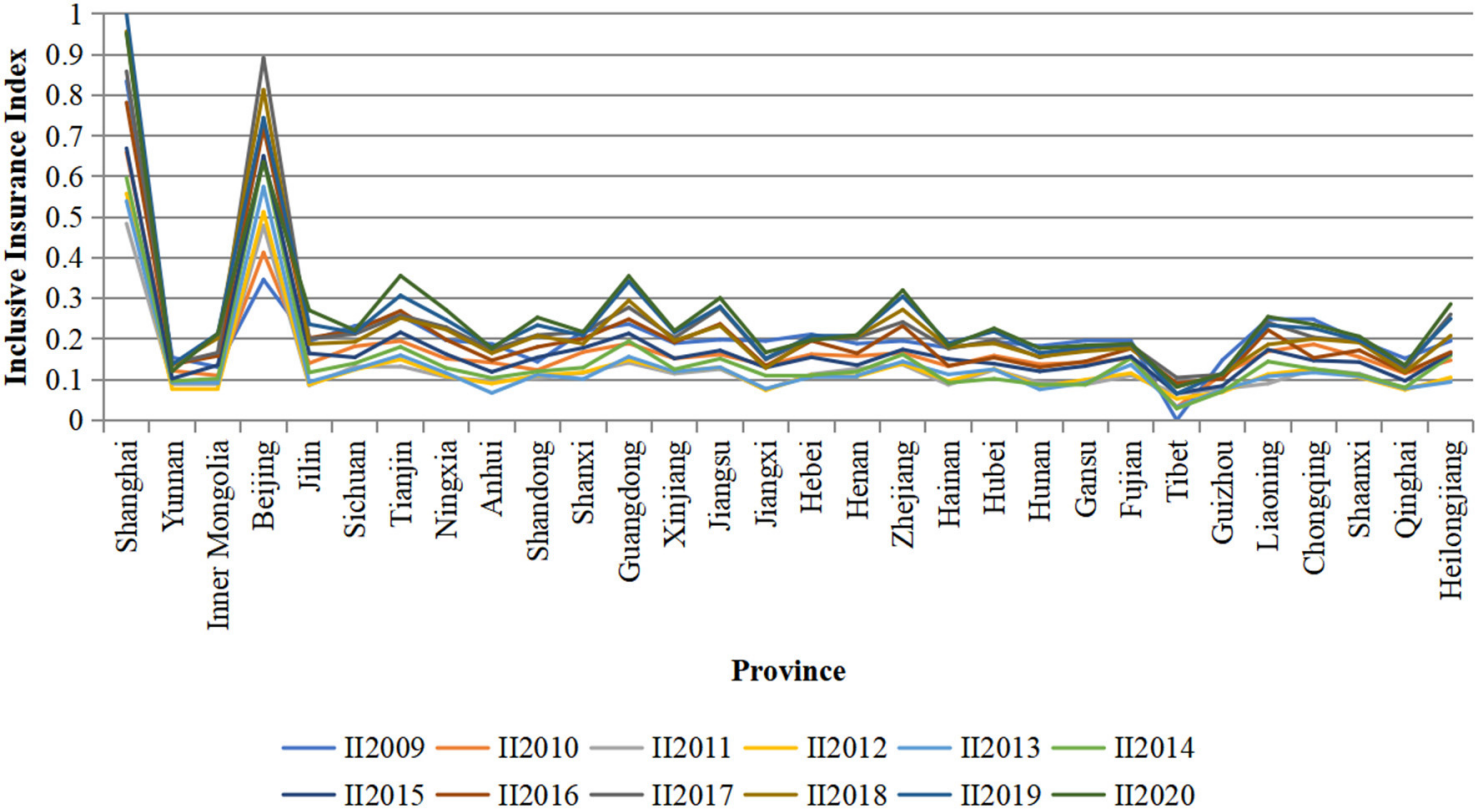
Inclusive insurance index of 31 provinces in China from 2009 to 2020.

Figure 1 shows that the development of inclusive insurance in China increased from 2009 to 2020. The median value of the digital inclusive financial index in all provinces in 2009 was 0.195, and it increased to 0.219 by 2020. Like most economic features in China, there are still some differences in the development degree of inclusive insurance between regions. The data for each year show that Beijing and Shanghai have the two highest inclusive insurance development indices, which are significantly higher than those of other regions and consistent with the economic environment of these two cities. The region with the lowest II development index is Tibet, followed by Guizhou. The stability of this index is illustrated by the fact that most regions have not changed much in the development degree of inclusive insurance. Moreover, it can be observed that the gap in inclusive insurance between regions is narrowing over time, which indicates that the universality of insurance has been greatly improved.

### Sample Description

Table 4 presents the descriptive statistics of the variables. The mean value of inclusive insurance is 0.21, and the standard deviation is 0.14. The mean value of income is 14,616.89, and the standard deviation is 3,0371.53. The mean value of the relative deprivation index is 0.41, and the standard deviation is 0.39. Variables with large variances, such as income, household assets, and liabilities, are taken as logarithms in the subsequent empirical analyses.

**Table 4:** Summary statistics.

**Variables**	**Average**	**Standard**	**Minimum**	**Maximum**	**Observations**
	**value**	**deviation**	**value**	**value**	
Inc	14,616.89	30,371.53	0	1,806,000	45,462
Rel	0.41	0.39	0	1	45,462
II	0.21	0.14	0.07	0.96	45,462
gen	0.48	0.50	0.00	1.00	45,462
age	40.78	11.71	18.00	60.00	45,462
mar	0.85	0.36	0.00	1.00	45,462
hou	0.74	0.43	0.00	1.00	45,462
edu	8.13	4.66	0.00	23.00	45,462
hea	2.84	0.98	1.00	5.00	45,462
emp	0.59	0.49	0.00	1.00	45,462
fin	215,536.10	810,358.70	1.00	10,000,000.00	45,462
deb	210,323.80	423,680.50	9.00	2,800,000.00	45,462
inf	4.18	1.09	1.00	5.00	45,462

### Baseline Estimates

First, the benchmark model is used to test the impact of inclusive insurance on income distribution. Table 5 reports the results based on Model (2).

**Table 5:** Baseline model results.

**Variables**	**Explained variables**	
	**Inc**	**Rel**
II	0.443^*^	−0.402^***^
	(1.857)	(−5.768)
gen	−0.669	−0.016
	(−1.405)	(−0.123)
age	0.024	−0.004
	(1.019)	(−0.712)
mar	0.037	−0.010
	(1.137)	(−1.107)
hou	0.024	−0.200^***^
	(0.692)	(−23.135)
edu	0.041^***^	−0.023^***^
	(3.514)	(−7.239)
hea	0.005	−0.003
	(0.372)	(−1.109)
emp	0.273^***^	0.048^**^
	(3.218)	(2.096)
fin	0.010^**^	0.000
	(2.207)	(0.019)
deb	−0.008^*^	−0.001
	(−1.852)	(−1.268)
inf	0.011	0.000
	(1.111)	(0.232)
Dev	0.051	0.176^***^
	(0.277)	(3.559)
Urb	0.015^**^	−0.021^***^
	(1.983)	(−10.468)
Fis	−0.065	0.226^***^
	(−0.516)	(8.103)
Str	−1.864^***^	1.490^***^
	(−2.707)	(8.517)
Tel	0.048	−0.012
	(0.745)	(−0.704)
Une	−0.052	0.126^***^
	(−1.229)	(11.873)
Intercept distance	8.376^***^	−2.441^***^
	(4.472)	(−4.603)
*N*	45,462	45,462
*R^2^*	0.128	0.331
*F*	37.839	549.909

*Standard errors are reported in parentheses*;

*,**, and *** *indicates significance at the 10, 5, and 1% levels, respectively*.

As shown in Table 5, the coefficient of the inclusive insurance index on income is positive and significant at the 10% level; that is, inclusive insurance significantly raises the income level. The coefficient of the effect of the inclusive insurance index on income deprivation is negative and significant at the 1% level; that is, inclusive insurance significantly decreases the relative deprivation index and thus has a positive effect on income distribution.

### Inclusive Insurance, Income Distribution, and Inclusive Growth

To examine whether inclusive insurance promotes inclusive growth, we use Model (3) to test the relationship of inclusive insurance, income distribution and inclusive growth. Table 6 lists the results.

**Table 6:** Inclusive insurance, income distribution, and inclusive growth.

**Variables**	**Inclusive growth model**
II	5.313^**^
	(2.010)
Income_it−1_	−0.328^***^
	(−5.549)
II_it_*Income_it−1_	−0.424^*^
	(standard error: −1.874)
Control variables	Join
Intercept distance	10.976^**^
	(2.142)
*N*	2,696.000
*R^2^*	0.328
*F*	13.403

*Standard errors are reported in parentheses*;

*,**, and *** *indicates significance at the 10, 5, and 1% levels, respectively*.

The results show that with the inclusion of the interaction term, the model fit increases to some extent, and the sign and significance of the estimated coefficients of the explanatory variables are also improved. When the explanatory variable is income, the coefficient of the interaction term of the inclusive insurance index and the income lagged term is significantly negative, indicating that groups with lower income levels benefit more from inclusive insurance. Inclusive insurance improves the effect of income distribution among the individuals within the sample to some extent. The results of the model verify the positive effect of inclusive insurance on inclusive growth. When the explanatory variable is the deprivation index, the coefficient of the interaction term of the inclusive insurance index and the lagged term of the deprivation index is significantly positive, indicating that groups with lower income levels benefit more from inclusive insurance and that inclusive insurance reduces the deprivation index of the sample to a certain extent.

### Subsample Test

Due to the differences in the development of inclusive insurance in different regions, urban and rural areas, as well as families of different income, sub sample analysis can better observe the heterogeneity of the impact of inclusive insurance on income distribution and inclusive growth.

#### Subsample Test by Regional

Some studies believe that agricultural insurance is generally beneficial for improving farmers' income (34, 75). However, there is regional heterogeneity in this effect (76), and the development level of inclusive insurance in China shows a distribution characteristic of “high in the east and west and low in the middle” (33). Based on the criteria of China's National Bureau of Statistics for classifying economic regions, the sample areas were divided into eastern, central and western regions for testing.

Comparing the results of the samples in the eastern, central and western regions (Table 7), we see that the impact of the inclusive insurance index on the income of the subsample is not significant. However, this impact increases gradually from the eastern region to the western region. The impact of the inclusive insurance index on Rel in the eastern region is not significant; in the central and western regions, it is significant at the confidence levels of 10% and 1%, indicating that this impact also increases gradually from the eastern region to the western region and is especially significant in the western region.

**Table 7:** Regional variation analysis.

**Variables**	**Eastern**		**Central**		**Western**	
	**Inc**	**Rel**	**Inc**	**Rel**	**Inc**	**Rel**
II	0.410	0.216	1.801	−1.101^*^	4.650	−0.490^***^
	(0.987)	(0.681)	(1.350)	(−1.858)	(1.495)	(−4.304)
Intercept	6.250^*^	0.777	8.661	−3.991^***^	12.896^*^	−3.126^*^
	(1.714)	(0.765)	(1.223)	(−3.132)	(1.793)	(−1.816)
*N*	9,046.000	18,932	5,566.000	13,389	3,988.000	13,141
*R^2^*	0.139	0.277	0.161	0.290	0.118	0.477
*F*	20.756	174.139	14.313	132.756	6.263	292.703

*Standard errors are reported in parentheses*;

* and *** *indicates significance at the 10 and 1% levels, respectively*.

#### Subsample Test by Urban-Rural

The empirical results of the urban-rural subsample test (Table 8) show that the impact of inclusive insurance is greater in rural areas than in urban areas. First, the impact of the inclusive insurance index on income is highly significant in rural areas but not significant in urban areas. Second, the impact of inclusive insurance on Rel is significant in both urban and rural areas, but the impact is higher in rural areas than in urban areas. The development of inclusive insurance is conducive to narrowing regional and urban-rural differences and promoting China's inclusive growth.

**Table 8:** Analysis of urban-rural differences.

	**Urban**		**Rural**	
**Variables**	**Inc**	**Rel**	**Inc**	**Rel**
II	0.244	−0.506^***^	0.986^***^	−8.527^***^
	(0.661)	(−5.650)	(2.622)	(−29.476)
Intercept	2.464	−2.235^***^	9.934^***^	−25.993^***^
	(1.082)	(−2.690)	(3.919)	(−28.263)
Control variables	Yes	Yes	Yes	Yes
*N*	6,724.000	22,014	11,876	23,448
*R^2^*	0.192	0.279	0.095	0.297
*F*	27.911	181.200	16.417	268.620

*Standard errors are reported in parentheses*;

*** *indicate significance at the 10% levels*.

#### Subsample Test by Household Income

The sample was divided into two groups based on income level, high-income and low-income groups, and the differences between the groups were separately tested.

The results of Table 9 show that inclusive insurance significantly raises the income level of low-income households, but the effect on the high-income group is not significant. The effect of inclusive insurance on Rel is significant for both groups and more significant for low-income households.

**Table 9:** Income gap analysis.

**Variables**	**High income group**		**Low-income group**	
	**Inc**	**Rel**	**Inc**	**Rel**
II	0.224	−1.626^***^	0.398^*^	0.527^*^
	(1.491)	(−22.084)	(1.679)	(1.665)
Intercept distance	8.602^***^	−9.769^***^	8.144^***^	1.270^**^
	(6.576)	(−28.305)	(4.396)	(2.136)
*N*	16,487	16,487	18,436	18,436
*R^2^*	0.203	0.097	0.131	0.628
*F*	54.966	133.753	38.243	9.226

*Standard errors are reported in parentheses*;

*,**, and *** *indicates significance at the 10, 5, and 1% levels, respectively*.

## Robustness Tests

In this section, we explore the robustness of our results to PSM-DID test, which is for reflecting the net effect of the inclusive insurance policy. We also use alternative measure of inclusive insurance by PKU-DFIIC and alternative estimation (GMM model) that handle the potential impact of outliers. Specifically, we construct panel threshold model to infer structural validity from coefficient robustness and plausibility.

### PSM-DID Test

Considering that the development level of the central and western areas is lower than that of the eastern area, the Chinese state has adopted a differential central fiscal subsidy policy for agricultural insurance for the sake of balanced development between regions. Taking the “Measures for the administration of agricultural insurance premium subsidies from the Central Treasury” promulgated in 2016 as an example, it is observed that the central financial administration's subsidy ratio for agricultural insurance for planting in central and western provinces is 5% higher than that in eastern provinces; additionally, agricultural insurance for farming is 10% higher (31). The “Measures for the administration of agricultural insurance premium subsidies from the Central Treasury” can be seen as a policy system with the concept of inclusive benefits, providing a “quasi-natural experiment” for this study. Therefore, in this section, we use 2016 as the time point for policy observation, regard the central and western regions as samples affected by the policy, and use the DID model. The impact of the policy is tested by comparing the changes in the differences between the samples affected by the policy and the samples not affected by the policy before and after the experiment (77, 78). Since the main beneficiary group of the policy is farmers, this section mainly selects rural residents, and the model is constructed as follows.


(7) $$\begin{array}{l} \begin{array}{c} Rel_{it}=\alpha_{0}+\alpha_{1}\text{I}{\text{I}}_{\text{it}}+\alpha_{2}\text{Treate}{\text{d}}_{it}+\sum_{k=1}^{N}\alpha_{k}X_{kit}\\ \;+\emptyset_{i}+\varphi_{i}+\mu_{it} \end{array} \end{array}$$

where Treated_it_ is the treatment variable. The “Measures for the administration of agricultural insurance premium subsidies from the Central Treasury” policy is used as the exogenous shock, and this variable takes the value of 1 when the year is ≥2016 and 0 otherwise. The coefficient α_2_ is the DID estimator, which portrays the causal impact of the policy on income distribution. *X*_*kit*_ is the set of control variables, andε_*it*_ denotes the random disturbance term. The model test results are as follows.

The results of the DID test are detailed in Table 10. PSM-DID reflects the net effect of the inclusive insurance policy. The interaction term has a significantly positive effect on household income and a negative effect on Rel that is significant at the 1% confidence level, indicating that the inclusive insurance policy has a significant positive effect on income distribution.

**Table 10:** Results of the DID test.

**Variable name**	**Inc**	**Rel**
II	0.905^***^	−0.436^***^
	(24.258)	(−44.762)
PSM-DID	0.612^***^	−0.143^***^
	(26.787)	(-34.012)
Intercept	7.363^***^	0.734^***^
	(122.991)	(84.309)
Control variables	Yes	Yes
*R* ^2^	0.361	0.267
Adjustment *R*^2^	0.361	0.267
*N*	12,514	69,594

*Standard errors are reported in parentheses*;

*, **, and *** *indicates significance at the 10, 5, and 1% levels, respectively*.

### Alternative Measure of Inclusive Insurance

To test the robustness of the results, the Peking University Digital Financial Inclusion Index of China^2^ (PKU-DFIIC) was used as a proxy for the explanatory variables.

Table 11 shows the results of using the PKU-DFIIC as the explanatory variable. The PKU-DFIIC significantly increases household income and decreases Rel, thus proving the reliability of the inclusive insurance index designed and indicating that the previous results are robust.

**Table 11:** Model results using the PKU-DFIIC as a proxy variable.

**Explanatory variables**	**Explained variables**	
	**Inc**	**Rel**
PKU-DFIIC	0.001***	−0.001***
	(3.850)	(−17.320)
Control variables	Yes	Yes
Intercept	6.133***	0.983***
	(23.361)	(4.110)
*N*	18,951	46,670
*R^2^*	0.125	0.335
*F*	101.828	1,096.056

Standard errors are reported in parentheses; The ^*^, ^**^, and ^***^ symbols indicate significance at the 10, 5, and 1% levels, respectively.

### GMM Test With Instrumental Variables

The endogeneity of inclusive insurance comes from several sources: first, reverse causality, where the level of household income affects the availability of insurance; second, omitted variables, where the development of inclusive insurance and household income may be influenced by other factors. Although the model incorporates a rich set of control variables to minimize the interference of omitted variables, there are still some variables that are unobservable. The third source is measurement error. The inclusive insurance index in this paper is a composite measure based on three dimensions and is generated by factor analysis, but there may still be bias. The use of alternative indicators also alleviates concerns about measurement error and reverse causality. To further test the robustness of the results, we test the causal relationship between inclusive insurance and income distribution and inclusive growth by introducing an instrumental variable approach.

The instrumental variable selected is individual risk preferences. We construct the level^3^ of individual risk preferences through a series of risk tests conducted by the CFPS. The obtained risk preferences are ordered discrete variables ranging from 0 to 5. The larger the value of risk preferences is, the more risk the individual prefers, and residents' risk preferences are highly correlated with the choice of insurance, satisfying the correlation conditions of instrumental variables.


(8) $$\begin{array}{l} Rel_{it}=\beta_{0}+\beta_{1}\text{I}{\text{I}}_{\text{it}}+\beta_{2}IV_{it}+\overset{N}{\underset{k=1}{\sum }}\beta_{k}X_{kit}+\emptyset_{i}+\varphi_{i}+\mu_{it} \end{array}$$

where Rel_it_ is the relative deprivation index, II_it_ is the level of inclusive insurance coverage, IV_it_ is an instrumental variable representing individual risk preferences, X_kit_ is the set of control variables, and μ_it_ is a random disturbance term.

To test the validity of the instrumental variables, under identification, weak instrument, and over identification tests were performed on the instrumental variables.

The results of the GMM model with the addition of instrumental variables (Table 12) show that the effect of II on income is significantly positive and that the effect on the deprivation index is negative and significant at the 1% confidence level, which is consistent with the results of the previous tests.

**Table 12:** Results of the GMM model with instrumental variables added.

**Variables**	**Explained variables**	
	**Inc**	**Rel**
II	1.253***	−0.263***
	(40.529)	(−31.731)
Instrumental variables	Yes	Yes
Control variables	Yes	Yes
Intercept distance	8.186***	−0.263***
	(168.974)	(−31.731)
N	37,421	69,594
Arellano–Bond test	−6.31	−52.34
Hansen test	340.10	948.11

Standard errors are reported in parentheses; The ^*^, ^**^, and ^***^ symbols indicate significance at the 10, 5, and 1% levels, respectively.

### Panel Threshold Model

Research found that the impact of agricultural insurance on farmers' income is non-linear and that agricultural insurance has a significant positive impact on farmers' income only when their income level crosses the threshold value and is at a higher stage (79). To further explore the characteristics of the inhibitory effect of inclusive finance on the income gap, we try to find whether there is a critical value of inhibitory relationship mutation and how to objectively judge this critical value through a panel threshold model. Family income is taken as the threshold variable to determine the number and size of the threshold value and to investigate whether there is an interval effect on the impact of the development degree of universal inclusive insurance on income deprivation. The specific model is as follows:


(9) $$\begin{array}{l} \begin{array}{c} Rel_{it}=\beta_{0}+\beta_{1}II_{it}\left(Income_{it}\leq \gamma \right)+\beta_{2}II_{it}\left(Income_{it}>\gamma \right)\\ \;+\sum_{k=1}^{N}\beta_{k}X_{kit}+\varepsilon_{it} \end{array} \end{array}$$

where Rel_it_ is the relative deprivation index, II_it_ is the level of inclusive insurance coverage, *Income*_*it*_ is household income, which is the threshold variable, γ is the threshold value to be estimated, X_kit_ is the set of control variables, and ε_*it*_ is a random disturbance term.

We first conduct a preliminary test on whether there is a threshold effect between the two. Bootstrapping is used to obtain the significance level of the test statistics, which is the main basis for judging whether there is a threshold effect between the two. Table 13 lists the results.

**Table 13:** Threshold effect test.

**Single threshold model**			**Double threshold model**		
**Estimated value**	***F*** **statistic**	***P***	**Estimated value**	***F*** **statistic**	***P***
0.25	33.77	0.036	0.18	8.37	0.356
Crit10	Crit5	Crit1	Crit10	Crit5	Crit1
25.35	31.31	38.24	15.49	18.62	30.19

The threshold value of the insurance index is 0.25 in the single threshold model, and the threshold value of the insurance index is 0.18 in the double threshold model. The threshold effect test *P* = 0.036 in the single threshold model and *P* = 0.356 in the double threshold model, indicating that there is a threshold effect at the 1% significance level in the single threshold model. Therefore, we consider that there is a single threshold effect of the impact of the inclusive insurance index on income distribution, and the single threshold effect model is chosen to analyze the empirical model. The results are shown in Table 14.

**Table 14:** Threshold regression results.

**Variables**	**Regional income level**	
	**Single threshold model**	
II	0	1
	−1.348	−1.524
Instrumental variables	Yes	Yes
Control variables	Yes	Yes

Standard errors are reported in parentheses; The ^*^, ^**^, and ^***^ symbols indicate significance at the 10, 5, and 1% levels, respectively.

The results show that for the threshold effect model, the elasticity coefficient of the effect of inclusive insurance development on income is −1.348 when II is below the threshold value of 0.250 and is significant at the 1% level. When inclusive insurance development is in this interval, inclusive insurance makes a significant contribution to income; when inclusive insurance development exceeds the threshold value, the elasticity coefficient becomes −1.524, indicating that the promoting effect of inclusive insurance on income increases at this time.

## Conclusion

We find that China's inclusive insurance developed rapidly from 2009 to 2020, showing regional differences, with regions such as Beijing and Shanghai experiencing faster insurance development but regions such as Tibet experiencing slower development. It can be observed that the gap in inclusive insurance between regions is narrowing over time, indicating that the universality of insurance has been greatly improved. The benchmark model show that the coefficient of the impact of the inclusive insurance index on income is positive and significant at the 10% level, indicating that inclusive insurance significantly raises the income level. Additionally, the coefficient of the impact of the inclusive insurance index on income deprivation is negative and significant at the 1% level, which means that inclusive insurance has a significant negative impact on income deprivation and reduces the relative deprivation index, thus having a positive impact on income distribution. We also show that groups with lower income levels benefit more from inclusive insurance and that inclusive insurance improves the effect of income distribution among individuals within the sample to a certain extent, verifying the positive effect of inclusive insurance on inclusive growth. The results of the subsample test indicate that the effect of inclusive insurance on income distribution gradually increases from east to west. The effect of inclusive insurance is greater in rural areas than in urban areas. Inclusive insurance significantly raises the income level of low-income households and has a more significant effect on the relative deprivation index of low-income households. The results of the PSM-DID test on the implementation of the “Measures for the administration of agricultural insurance premium subsidies from the Central Treasury” indicate that the inclusive insurance policy has a significant positive impact on income distribution. The robustness of the results is also demonstrated by using the Peking University Digital Financial Inclusion Index of China (PKU-DFIIC) as the alternative variable and the GMM model with instrumental variables. The results of the threshold effect model show that when the value of the inclusive insurance index is below the threshold value of 0.250, the elasticity coefficient of the effect of the development of inclusive insurance on income is −1.348 and is significant at the 1% level. Our results indicate that when the development of inclusive insurance is in this range, inclusive insurance makes a significant contribution to income. Additionally, when the value of the inclusive insurance index exceeds the threshold value, the elasticity coefficient becomes −1.524, indicating that inclusive insurance makes a significant contribution to income and the contribution of inclusive insurance to income increases.

Based on the conclusions, we propose the following policy recommendations: First, there are great differences in the development of different regions in China; thus, we should adapt to regional characteristics, flexibly and reasonably design the inclusive insurance system, carry out inclusive insurance based on local conditions, reduce the cost of insurance service and expand the service radius in view of the unbalanced development of universal insurance between regions. Second, government should promote the development of inclusive insurance, improve the risk management ability of insurance institutions, reduce their operating costs, make inclusive insurance better serve the policy target groups, and effectively disperse and protect their production and life risks.

There are still some expansions in our study: firstly, we only calculate the inclusive insurance index by provinces in China limited by the availability of data. If more data can be obtained in the future, we can calculate China's inclusive insurance index in cities or counties. Secondly, vigorously developing inclusive finance with the concept of inclusiveness is of great significance to improve income distribution and economic development, and more attention should be paid to regional balance and institutional conditions.

Obviously, the development potential of inclusive insurance is greater in countries that do not have a developed financial industry or sophisticated products. Among countries where microinsurance is offered or being introduced, possibly only South Africa could be considered to be a mature conventional insurance market. A mature market may be defined as one that has passed both the emerging and growth phases of the industry. Earnings and sales grow tend to be slower in mature markets and there may be a lack of significant innovation. In contrast, most microinsurance markets are still emerging: microinsurance has expanded rapidly in India, and is growing or diversifying on the foundation of developing insurance mark in South America and Asia, whereas in Africa, microinsurance is relatively nascent. In the future, we can further explore the mechanisms through which inclusive insurance affects income distribution and inclusive growth, and study policy measures to achieve inclusiveness in these countries.

## Data Availability Statement

Publicly available datasets were analyzed in this study. This data can be found at: http://www.isss.pku.edu.cn/cfps/.

## Author Contributions

LZ: design of the paper, methodology, analysis of data, drafting the paper, review & editing, and responsible for ensuring that the descriptions accurate and agreed by YS. YS: collect the data, data curation, and translate. All authors contributed to the article and approved the submitted version.

## Funding

We gratefully acknowledge the financial support of the National Natural Science Foundation of China (Grant No. 71903209).

## Conflict of Interest

The authors declare that the research was conducted in the absence of any commercial or financial relationships that could be construed as a potential conflict of interest.

## Publisher's Note

All claims expressed in this article are solely those of the authors and do not necessarily represent those of their affiliated organizations, or those of the publisher, the editors and the reviewers. Any product that may be evaluated in this article, or claim that may be made by its manufacturer, is not guaranteed or endorsed by the publisher.

## Footnotes

^1^Data Source: China Banking and Insurance Regulatory Commission, 2021.

^2^The PKU-DFIIC covers 31 provinces (municipalities, autonomous regions), 337 cities (regions, autonomous prefectures, leagues, etc.) and ~2,800 counties (county-level cities, banners, municipal districts, etc.) in mainland China. Among them, municipal district data were not available in 2014–2015.

^3^Risk preference was determined based on questions QN101-QN105 in the individual self-response questionnaire that asked the respondents about their behavioral and mental status, and the degree of risk preference was measured by the answers that respondents ultimately stopped selecting based on the risk test flowchart.
